# What Goes In, Must Come Out: Generative Artificial Intelligence Does Not Present Algorithmic Bias Across Race and Gender in Medical Residency Specialties

**DOI:** 10.7759/cureus.54448

**Published:** 2024-02-19

**Authors:** Shu Lin, Saket Pandit, Tara Tritsch, Arkene Levy, Mohammadali M Shoja

**Affiliations:** 1 Department of Medical Education, Nova Southeastern University Dr. Kiran C. Patel College of Allopathic Medicine, Fort Lauderdale, USA

**Keywords:** bias identification, medical education, healthcare, diversity, artificial intelligence

## Abstract

Objective

Artificial Intelligence (AI) has made significant inroads into various domains, including medicine, raising concerns about algorithmic bias. This study investigates the presence of biases in generative AI programs, with a specific focus on gender and racial representations across 19 medical residency specialties.

Methodology

This comparative study utilized DALL-E2 to generate faces representing 19 distinct residency training specialties, as identified by the Association of American Medical Colleges (AAMC), which were then compared to the AAMC's residency specialty breakdown with respect to race and gender.

Results

Our findings reveal an alignment between OpenAI's DALL-E2's predictions and the current demographic landscape of medical residents, suggesting an absence of algorithmic bias in this AI model.

Conclusion

This revelation gives rise to important ethical considerations. While AI excels at pattern recognition, it inherits and mirrors the biases present in its training data. To combat AI bias, addressing real-world disparities is imperative. Initiatives to promote inclusivity and diversity within medicine are commendable and contribute to reshaping medical education. This study underscores the need for ongoing efforts to dismantle barriers and foster inclusivity in historically male-dominated medical fields, particularly for underrepresented populations. Ultimately, our findings underscore the crucial role of real-world data quality in mitigating AI bias. As AI continues to shape healthcare and education, the pursuit of equitable, unbiased AI applications should remain at the forefront of these transformative endeavors.

## Introduction

The past decade has witnessed a remarkable surge in tools using artificial intelligence (AI). This technology has effectively reduced the time spent on menial tasks, thus improving the quality of our daily lives [1]. From customer support interactions through phone lines and online chats to the advent of food delivery robots and the emergence of self-driving cars, the realm of AI is expanding exponentially. It is inevitable that AI will soon be integrated more significantly in domains such as medicine, and education [2]. Yet, a fundamental question looms: does AI’s impact elevate all lives equitably, or does it unconsciously tilt toward certain groups, perpetuating societal disparities? In the realm of AI, bias can manifest in two distinct forms. First, the algorithmic AI bias, or "data bias," arises from training algorithms with partial or skewed data, leading to outputs that cannot be generalized [3]. Second, societal AI bias can arise from cultural assumptions and norms that can create blind spots and preconceived expectations in the AI responses [4].

The risk of AI bias needs to be recognized and addressed or it has potential to worsen systemic racial and sex disparities if it is used on a large scale [5,6]. Recent studies have shown that AI can develop bias in multiple settings [7,8]. In a study by Caliskan *et al*., a word-embedding association test was created to determine the strength of the association between words and the association between those in the real world [9]. They found that there was a high degree of correlation between the biases possessed by the AI that they had created and existing human biases. Especially problematic toward race or gender, one finding from the study was that a bundle of names associated with being “European American” was found to be significantly more easily associated with pleasant than unpleasant terms, compared to a bundle of “African American” names. It indicates that the bias present in AI results from the bias in the given data. Another study by Obermeyer *et al*. investigated AI bias in healthcare settings. They found that a clinical algorithm used by hospitals that decided which patients needed care exhibited racial bias [10]. The AI determined that black patients had to be much sicker than white patients to be recommended for the same care. This was due to previous data showing that black patients overall have lower healthcare expenditure than white patients, and therefore needed to be much sicker to receive the same type of care. This is an example of how AI has the potential to worsen the existing inequalities in our society. A study conducted by Makhortykh *et al*. also explored the presence of race and gender bias in AI image search results from six different search engines [11]. Their findings revealed that these search engines tend to prioritize anthropomorphic AI images featuring white faces.

In the current world, bias exists in medicine, especially in the different fields of medicine. There are gender gaps in medical specialties, with male-dominated specialties such as neurosurgery, orthopedic surgery, and general surgery, and also female-dominated specialties such as family medicine, pediatrics, and obstetrics and gynecology [12-14]. Moreover, despite women comprising just over 50% of the population of the United States, they constitute just 36% of physicians [15]. These discrepancies have also been noted in academic medicine, in which the number of women in faculty positions has steadily been increasing but stagnates at higher positions [16]. This unequal representation could be due to decreased opportunities for professional advancement based on gender and race [17]. It could also be due to institutional policies that benefit a certain population more than another. The goal of this study is to investigate whether AI has learned from this skewed representation of the medical field, and in return, also associates medical specialties with certain race and gender attributes.

The present study examines the biases related to sexual orientation and race within Generative Artificial Intelligence (GAI) across 19 distinct medical specialties. In the existing healthcare landscape, a significant gender imbalance is evident, with a preponderance of males in high-demand areas like surgery, while less demanding fields such as family medicine and pediatrics showcase a more balanced gender distribution. This study seeks to ascertain whether AI has assimilated such representations of medical specialties, possibly leading to the attribution of certain gender or ethnic predominance to specific fields based on its training data. The primary objective is to evaluate the extent to which AI may have internalized existing biases concerning gender and ethnicity within the context of medical specializations.

## Materials and methods

DALL-E2, a cutting-edge GAI technology developed by OpenAI (San Francisco, U.S.), has demonstrated its remarkable ability to craft lifelike images and artistic representations based on user-provided prompts. We prompted DALL-E2 to generate facial representations specifically tailored to 19 distinct medical specialties delineated by the American Academy of Medical Colleges (AAMC) [18]. This diverse array of specialties encompasses urology, diagnostic radiology, radiation oncology, psychiatry, physical medicine and rehabilitation, pediatrics, pathology, otolaryngology, orthopedic surgery, ophthalmology, obstetrics and gynecology, neurological surgery, neurology, internal medicine, general surgery, family medicine, emergency medicine, dermatology, and anesthesiology. To ensure impartiality and mitigate linguistic bias, concise descriptions were crafted for each specialty. For every specialty, the DALL-E2 model was engaged to generate a quartet of images. We prompted DALL-E2 independently on three occasions for each specialty's description input, thereby engendering a collection of 12 distinctive images per specialty. Any instances of AI-generated images in a cartoonesque format were intentionally omitted from our analysis due to the inherent challenges in accurately ascertaining nuances of gender and race within such depictions.

Two reviewers analyzed the AI-generated images, individually assigning gender and race to each image. This process was executed independently to ensure internal reliability. In cases where disparities emerged, a third reviewer was introduced to arbitrate and reconcile differences. Ultimately, gender and race attributes for each image were established based on a consensus reached by a minimum of two out of the three reviewers. The images were categorized into binary gender classifications (male and female), but only four racial categories (Asian, Black or African American, Hispanic or Latino, or of Spanish origin, and white) were established due to limitations in identifying a more diverse range of races by the reviewers. Subsequently, the AI-generated images corresponding to each specialty were juxtaposed against the gender and race distribution within their respective specialties as outlined in the AAMC's 2021-2022 report [19,20].

Statistical analysis

The statistical analysis was performed by looking at two different categorical variables: gender and ethnicity. The proportions were compared against one another to determine the statistically significant differences via a two-proportion Z-test. This workflow was repeated across each of the different specialties. As an example of this workflow, the proportion of anesthesiologists generated by DALL-E2 who were female was compared against the proportion of anesthesiology residents who were female as reported by the AAMC data. This process was repeated for all specialties under consideration. Bonferroni-adjusted significance levels ($\alpha$) were used to control the number of multiple paired comparisons and to reduce type I errors. As there were 19 different specialties under consideration, $&alpha;=0.05/19&asymp;0.0026$. All statistical analyses were performed via R (version 4.3.1, R Foundation for Statistical Computing, Vienna, Austria).

## Results

The demographic profiles of incoming residents, as reported by the AAMC, were juxtaposed with the demographics of AI-generated images (Table 1).

**Table 1:** Racial and gender distribution data across 19 different medical specialties comparing AI-generated faces with the AAMC report. AA, African American; AI, Artificial Intelligence; AAMC, Association of American Medical Colleges.

	Race										Gender					
	AI					AAMC					AI			AAMC		
Specialty	White	AA	Asian	Hispanic	Total	White	AA	Asian	Hispanic	Total	Men	Women	Total	Men	Women	Total
Anesthesiology	10	0	1	1	12	2801	352	1283	413	4849	8	4	12	4345	2255	6600
Dermatology	11	0	0	1	12	804	74	332	98	1308	0	12	12	593	920	1513
Emergency medicine	6	1	3	2	12	4129	424	941	618	6112	9	3	12	5249	3409	8658
Family medicine	5	3	4	0	12	4434	936	1921	1088	8379	6	6	12	6420	7773	14193
Internal medicine	3	3	4	2	12	8205	1250	5789	1785	17029	7	5	12	16468	13096	29564
Neurological surgery	11	0	1	0	12	893	71	350	113	1427	10	2	12	1231	337	1568
Neurology	9	0	1	2	12	1123	116	582	223	2044	7	5	12	1689	1554	3243
Obstetrics and gynecology	12	0	0	0	12	2960	483	786	471	4700	0	12	12	778	4950	5728
Ophthalmology	10	0	1	1	12	720	46	420	96	1282	4	8	12	832	605	1437
Orthopedic surgery	7	1	4	0	12	2722	215	518	269	3724	8	4	12	3542	792	4334
Otolaryngology	11	0	1	0	12	1012	66	389	103	1570	4	8	12	1033	698	1731
Anatomic and clinical pathology	8	2	2	0	12	804	92	387	137	1420	7	5	12	1097	1176	2273
Pediatrics	9	1	0	2	12	3998	515	1305	735	6553	4	8	12	2481	6738	9219
Physical medicine and rehabilitation	4	2	5	1	12	504	66	253	84	907	8	4	12	981	513	1494
Psychiatry	10	0	1	1	12	2907	486	1308	573	5274	7	5	12	3495	3598	7093
Radiation oncology	9	0	3	0	12	390	37	216	36	679	4	8	12	501	250	751
Diagnostic radiology	10	0	2	0	12	1985	164	942	253	3344	8	4	12	3126	1200	4326
General surgery	8	1	3	0	12	4961	554	1557	773	7845	9	3	12	5314	4540	9854
Urology	9	0	1	2	12	1016	86	380	135	1617	9	3	12	1237	543	1780

**Figure 1:**
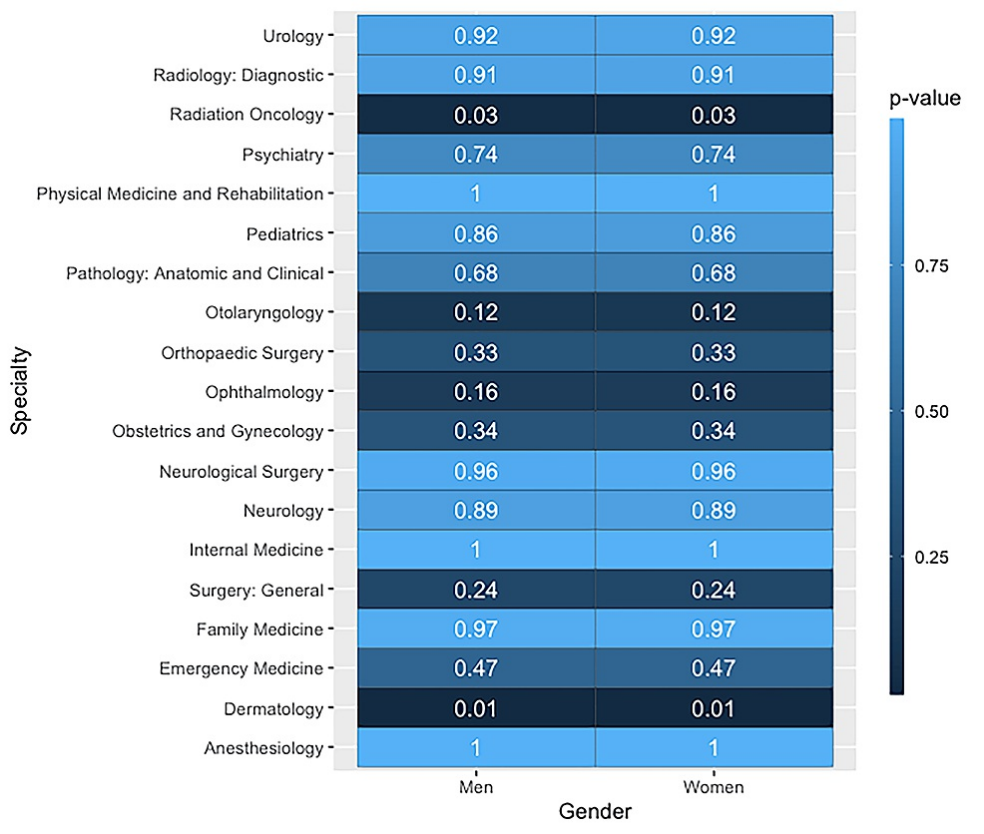
Heatmap of P values from two-proportion Z-tests, comparing proportions of each gender across 19 different medical specialties. At this level, there appear to be no statistically significant differences between the two datasets used for this analysis.

An examination of gender distribution exhibited no statistically significant discrepancies between the two datasets, as attested by Bonferroni-adjusted confidence levels. The summary of the gender-based analysis is presented in Figure 1.

**Figure 2:**
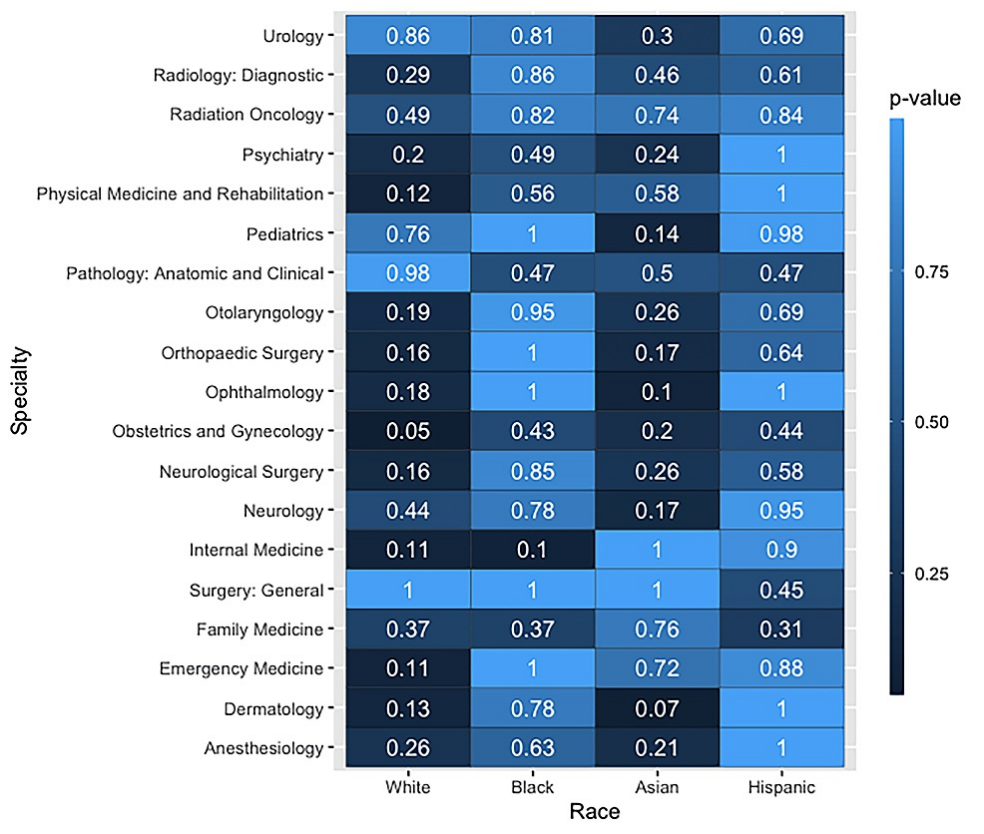
Heatmap of P values from two-proportion Z-tests, comparing proportions of ethnicities across 19 different medical specialties. At this level, there appears to be no statistically significant differences between the two datasets used for this analysis. Greyed-out boxes represent ethnic groups with insufficient representation in the AI-generated dataset.

Similarly, the analysis by ethnicity also demonstrated no significant differences between the AAMC data and the ethnic makeup of AI-generated faces after the application of Bonferroni-adjusted confidence levels. The summary of the analysis by ethnicity is presented in Figure 2.

## Discussion

In recent years, AI has found more applications across various domains, including the medical field. This study aimed to investigate potential biases related to sexual orientation and racial representation in a popular GAI system (OpenAI's DALL-E2), within the context of 19 different medical residency specialties. Our primary goal was to assess whether the AI system tends to associate gender and race with specific medical fields. Our results revealed that DALL-E2's predictions regarding the representation of genders and races in each medical specialty closely aligned with the demographic data provided by the AAMC for the 2021-2022 residency programs. Notably, DALL-E2 consistently attributed male dominance to medical specialties known for their high demands, such as neurosurgery, orthopedic surgery, and urology. This observation corresponded with the AAMC's existing resident data, which indicated a preponderance of males in these specialties. These findings suggest that the biases observed in DALL-E2 may be reflective of the training data used to develop the AI system rather than inherent algorithmic biases.

Recent studies have extensively explored gender disparities within various medical specialties. Despite efforts to address gender biases, one study spanning two decades in academic medical centers showed that little progress has been made in transforming predominantly male-dominated fields, and women physicians continue to face significant challenges in these domains [21,22]. One study explored the potential model bias that emerges when training AI models exclusively on one gender dataset for predicting early-stage severity based on COVID-19 patient medical records [23]. They found that gender-dependent AI models exhibited lower accuracy compared to unbiased models, underscoring the importance of training models on unbiased data. Unfortunately, the data used to train AI models often suffer from skewed demographics, primarily favoring male representation. One study found that even gender-neutral internet searches yield male-dominated results due to the societal biases ingrained in the training data [24]. Furthermore, natural language processing (NLP), a key component of certain AI systems like Apple's Siri and Amazon's Alexa, has also been shown to harbor gender bias [25]. These systems tend to associate 'man' with 'doctor' and 'woman' with 'nurse,' a reflection of historical data rather than the contemporary reality. However, our study's findings regarding the gender and race distribution in medical specialties align with the current demographic landscape. This alignment highlights a critical issue, gender bias within specific medical specialties contributes to biased outputs in AI systems, further perpetuating the existing gender disparities in these fields.

Our study also reveals an alignment between our findings and the racial representations in various medical specialties, as reported by AAMC. Medical fields such as neurosurgery, radiology, and dermatology, which are predominantly composed of white practitioners, exhibited the highest representation of white individuals in our GAI-generated data. Conversely, medical specialties like emergency medicine, family medicine, and internal medicine demonstrated greater racial diversity in GAI-generated data, consistent with contemporary AAMC data. The presence of racial bias in AI systems is a well-documented concern. Historically, facial analysis software struggled to accurately detect dark-skinned faces, often requiring individuals to wear a white mask to be accurately recognized [26]. This was because the system was trained on images of men with predominantly fair skin. Another study examined an algorithm employed to predict healthcare needs for a large patient population, revealing a bias against black patients based on healthcare spending [27]. Due to historical disparities in access to care, black patients tend to spend less on healthcare, leading to recommendations for care only when their health conditions are significantly more severe compared to their white counterparts [27].

Evidence from several studies has suggested that racial discrepancies exist in different medical specialties [28,29]. Achieving diversity in racial representation within different fields of medicine holds significant importance. A study conducted by Cooper-Patrick and colleagues illuminated this concern by revealing that African American patients rated their interactions with physicians as less engaging compared to their white counterparts [30]. Interestingly, the same study found that patients often perceive physicians of their racial background as having a more engaging decision-making style [30]. This shows the importance of racial representation in all specialties to ensure care for patients from diverse ethnic backgrounds. These findings underscore the critical role of racial representation in all medical specialties. Ensuring diversity in healthcare professions is essential to providing equitable care for patients from diverse ethnic backgrounds. It not only enhances patient-provider interactions but also contributes to a more inclusive and culturally sensitive healthcare system.

Limitations

This study must be viewed in the context of its limitations. We only generated 12 images per specialty, which inherently constrains the level of representation achievable within our dataset. To mitigate potential bias in the data interpretation process, we employed a review process involving two independent reviewers who assessed all 12 images for each of the 19 specialties. Any discrepancies were resolved by a third reviewer. It is important to acknowledge that individual bias may exist among the reviewers, potentially influencing their attributions of race or gender to specific AI-generated features. Additionally, our study compared our dataset to a dataset consisting of all active residents, rather than the overall active physician demographics. We decided against including a broader physician population due to concerns regarding overfitting. Including all active physicians could have introduced redundancy in our analysis, as OpenAI's models are trained on a vast dataset of text-associated images available on the internet up to 2021. The overlap between our evaluation data and the training data could lead to overfitting, where a model's accuracy is falsely inflated due to using the same data for both training and validation. To address this issue, we opted to use the 2021-2022 active resident data, providing an independent validation dataset that more effectively assesses the biases of DALL-E2.

## Conclusions

This study examined the burden of algorithmic bias within OpenAI's DALL-E2 concerning gender and race across medical specialties. Our findings suggest the absence of algorithmic bias, as the specialist demographics it portrays align with the current profile of medical residents. However, this discovery raises critical ethical questions about the utilization of AI tools in medicine and healthcare. It is essential to acknowledge that our study should be considered a starting point for further research rather than definitive evidence of the absence of bias in AI. Our study underscores that AI excels at discerning patterns in the data it is fed. Yet, it is crucial to recognize that if the data carries underlying biases, the AI tool will inevitably perpetuate these biases. This revelation holds profound significance as AI increasingly integrates into the realms of medicine and education. To mitigate AI bias, we must address the biases that exist in the real world. By confronting these disparities, we can provide AI with more unbiased data to learn from and foster a more impartial worldview. Presently, the medical realm is undergoing a concerted drive toward inclusivity across races and genders. These initiatives are perceptible and are contributing to the transformation of medical education. However, the sustainability of this transformation at a national level remains to be seen. To forge an equitable path forward, continued efforts are imperative to dismantle barriers and foster inclusivity in historically white-male-dominated medical domains for underrepresented populations. The present study carries significant implications for the development of AI systems, particularly in the context of healthcare and beyond. It highlights the importance of meticulous data curation and diversity in training datasets. To create more inclusive and unbiased AI systems, we need to prioritize the collection of diverse and representative data. This entails actively addressing biases in data sources and ensuring that underrepresented populations are adequately represented. Furthermore, our study underscores the necessity of continuous monitoring and evaluation of AI systems in real-world applications. Regular audits and assessments of AI algorithms for fairness and equity should be integrated into AI development pipelines.
